# The changing role of natural killer cells in cancer metastasis

**DOI:** 10.1172/JCI143762

**Published:** 2022-03-15

**Authors:** Isaac S. Chan, Andrew J. Ewald

**Affiliations:** 1 Department of Internal Medicine, Division of Hematology and Oncology, and; 2 Harold C. Simmons Comprehensive Cancer Center, University of Texas Southwestern Medical Center, Dallas, Texas, USA.; 3 Department of Oncology, Sidney Kimmel Comprehensive Cancer Center, and; 4 Department of Cell Biology, Johns Hopkins University School of Medicine, Baltimore, Maryland, USA.

## Abstract

Natural killer (NK) cells are innate immune cells that are critical to the body’s antitumor and antimetastatic defense. As such, novel therapies are being developed to utilize NK cells as part of a next generation of immunotherapies to treat patients with metastatic disease. Therefore, it is essential for us to examine how metastatic cancer cells and NK cells interact with each other throughout the metastatic cascade. In this Review, we highlight the recent body of work that has begun to answer these questions. We explore how the unique biology of cancer cells at each stage of metastasis alters fundamental NK cell biology, including how cancer cells can evade immunosurveillance and co-opt NK cells into cells that promote metastasis. We also discuss the translational potential of this knowledge.

## Introduction

Immune-modulating therapies have revolutionized the field of oncology and extended survival in many cancer patients (1). Accordingly, preclinical research now emphasizes understanding the role of the tumor microenvironment in cancer progression. While T cells have been the focus of the first generation of clinically approved immunotherapies, emerging work has revealed diverse mechanisms regulating the tumor-suppressive and tumor-promoting qualities of other immune cell populations. Specifically, natural killer (NK) cells are lymphoid members of the innate immune system that have potent antitumor and anti-metastatic abilities (2, 3).

Since their identification in the 1970s, NK cells have been described as critical contributors to the immune control of cancer cells (4–15). NK cells are unique in that initiation of their cytotoxic function does not require prior exposure to tumor antigens (16). Furthermore, their presence in the peripheral blood correlates with better prognosis in melanoma (17), breast (18), prostate (19), renal cell (20), and colorectal cancers (21). These clinical observations led to the development of NK cell–based therapies, including transplanted donor NK cells, engineered “off-the-shelf” NK cells, and antibody blockade of inhibitory receptors on NK cells (22). While NK cells do not require prior tumor antigen exposure, they are regulated by diverse activating and inactivating receptors that regulate tumor recognition and cytotoxic activity (23–37). Cancer cells can use these receptors to alter NK cell function and reduce their cytotoxic activity (38, 39). There are multiple classes of inhibitory receptors that can diminish NK cell cytotoxicity, including killer immunoglobulin-like receptors (KIRs; ref. 40), T cell immunoreceptor with Ig and ITIM domains (TIGIT; refs. 41, 42), lymphocyte activation gene 3 (LAG3; ref. 43), killer cell lectin-like receptor subfamily G member 1 (KLRG1; refs. 44, 45), and NKG2A (46–48). NK cell–directed therapeutics are at early stages of clinical development but are being developed at a rapid pace (49).

Although NK cells have a potent ability to eliminate cancer cells in the primary tumor and at distant sites (50), every clinically significant tumor in a patient has somehow evaded this control. Indeed, recent work has shown that NK cells can even be co-opted to promote cancer progression (51). Therefore, there is an urgent need to understand how NK activity is regulated in peripheral organs and to identify strategies to recruit and sustain an NK cell–mediated antitumor response. Achieving this goal is more complex than getting NK cells to the right place: both cancer cells and the microenvironment are capable of inhibiting or co-opting NK activity. We anticipate that a deeper understanding of these signals and how they dynamically regulate NK cell activity will lead to novel therapeutic strategies.

In this Review, we highlight the recent body of work that has begun to answer how NK cells influence specific stages of metastasis and how the unique biology of metastatic cells alters NK cell function. The lessons learned from this growing body of work can help to improve NK cell–focused anticancer therapeutics.

## Dynamic biology of metastatic cancer cells

Metastasis is the major driver of cancer deaths (52). Metastasis itself is a multistep process that starts as cancer cells invade and disseminate out of the primary tumor, intravasate into and extravasate out of circulation, evade the immune system, and form new tumors in distant organs (53). The idea that NK cell activity varies in different organs and at different stages of metastasis makes perfect sense once we think about metastasis at the cell biological level. To accomplish these varied tasks, cancer cells must alter their shape, behavior, and molecular repertoire. These changes have consequences for tissue architecture, organ function, and cancer cell–immune interactions.

Successful progression through the metastatic cascade must account for the cancer cell–intrinsic and –extrinsic factors that limit the spread and outgrowth of metastatic seeds. One extensively studied cellular program that explains these metastatic behaviors is epithelial-mesenchymal transition (EMT). The EMT program is utilized by epithelial cancer cells to acquire characteristics that increase their success at metastasis. These features often correlate with mesenchymal traits that involve increased motility, increased invasiveness, and increased extracellular matrix degradation (54). Cells have also been identified as concurrently exhibiting epithelial and mesenchymal programs, described as discrete “partial” or “hybrid” EMT states or, alternatively, as varying positions on a continuum of epithelial-to-mesenchymal plasticity (55–57). In particular, there is increasing evidence that the EMT program is context dependent with multiple regulatory networks involved (58).

EMT has been the predominant model for conceptualizing metastasis by individual cancer cells (59). However, cancer invasion is frequently accomplished by adherent groups of cells (60), and these collective mechanisms can give rise to multiclonal metastases (61–63). Indeed, invasion of cancer cells out of the primary tumor can be accomplished by the migration of groups of cells that maintain their epithelial properties and intercellular adhesion (63–65). In particular, our laboratory discovered that the most invasive cells in a primary tumor are behaviorally and molecularly distinct from the bulk tumor (63). These cells expressed keratin-14 (K14) and p63 and led collective invasion in in vivo and in vitro models. They were also overrepresented in lung metastases. This study further established the requirement of K14 and p63 for collective invasion in primary cells (63).

The process of collective migration conveys survival advantages to clusters of cancer cells as they invade and disseminate from the primary tumor, enter circulation, and seed distant sites (66). Clinically, circulating cancer cell clusters have been found in a variety of tumors, including breast, brain, lung, prostate, renal esophageal, and melanoma, and have been shown to correlate with worse survival outcomes (67–70). And, as discussed further below, collective seeding of distant organs has been shown to increase metastatic efficiency. Collective migration and EMT are not mutually exclusive, and it has been observed that collectively migrating units can exhibit heterogeneous populations of cells with varying degrees of epithelial or mesenchymal traits (71, 72).

While the cytotoxic activity of NK cells has long been linked to anti-metastatic activity and the reduction of distant site metastases (73), recent studies have focused on the function of NK cells and their interactions at specific stages of metastasis.

## Disseminated cancer cells can evade NK cell surveillance

After leaving the primary tumor, metastatic cancer cells enter circulation. There is considerable interest in determining how metastatic seeding occurs and specifically in whether this process is driven by single cancer cells or by polyclonal clusters of cancer cells (74). Using preclinical models, several groups have shown that polyclonal clusters are more effective and efficient at both surviving circulation and establishing metastases at distant sites (61–63, 75). Lineage tracing analyses in animal models suggest that cancer cell clusters are more efficient at forming distant metastases than single cancer cells (61–63). Interestingly, as cancer cell clusters form macrometastases, they may lose some epithelial markers, like K14, but continue to require others, like E-cadherin (76). Cancer cell clusters can achieve these prosurvival properties by hypomethylating genes related to embryonic stem cells (77). However, most of these studies were performed in immunocompromised mice. An outstanding question remained: do polyclonal cancer cell clusters have advantages that help them resist the host’s immune system and specifically NK cells?

To address this question, Lo et al. used a transplant system to test the efficiency of monoclonal and polyclonal circulating cancer cell clusters (78). They engrafted either a mixed or single population of GFP- or mCherry-labeled mouse mammary cells into the mammary fat pad of the recipient mouse host. The hosts differed by their degree of immunodeficiency. Interestingly, they found that polyclonal clusters were more effective at forming metastatic lesions than single cells in immunocompetent WT mice and nude mice that lacked T cells. However, in NOD-Rag1^null^IL2rg^null^ (NRG) mice that lacked B, T, and NK cells, they observed an increase in the number of monoclonal metastatic lesions. Thus, in NRG mice, the contribution of polyclonal metastases to the overall number of lesions formed was reduced. Further, they found that depleting NK cells, but not macrophages, NKT cells, or T cells, shifted the ratio of monoclonal lesions to polyclonal lesions. This result suggested that NK cells are more effective in suppressing single, monoclonal seeds than clusters (78).

They then performed transcriptomic analysis on cluster-forming cancer cells that were resistant to NK cell cytotoxicity and non-cluster-forming cancer cells that were sensitive to NK cell cytotoxicity. This analysis revealed that, relative to cancer cells resistant to NK cell killing, cancer cells sensitive to NK cell killing had lower expression of genes related to the regulation of cell-cell adhesion and higher expression of genes encoding ligands that activate NK cell receptors. The authors hypothesized that the process of EMT correlates with sensitivity to NK cell cytotoxicity. In a series of elegant experiments either perturbing the “epithelial state” of cancer cells or performing additional analysis of metastatic lesions after the adoptive transfer of NK cells, they determined that the expression of ligands that activate NK cell cytotoxicity correlates closely with a lower epithelial state (78). Interestingly, it appears that this correlation occurs independently of NK cell selective pressure, suggesting that these programs are intrinsic to the metastatic cancer cell. Thus, the advantages of polyclonal clusters appear to be multifaceted: beyond the physical adhesion properties that make these clusters more efficient at metastatic seeding, alteration of epithelial or mesenchymal properties can make them less or more susceptible to NK cell killing.

The paper by Lo et al. (78) adds to initial observations that NK cells play a key role in clearing circulating cancer cells (79, 80). Recently, Sathe et al. used a model of circulating B16F10 melanoma cells to show that the disruption of Mcl1 on NK cells, a key protein critical to NK cell survival, results in increased cancer cell seeding at distant sites (81). Work is ongoing to clarify our understanding of the role NK cells play in regulating circulating cancer cells. What is beginning to emerge is that circulating cancer cells have unique properties distinct from the primary tumor and developing distant-site macrometastases. These cancer cell–intrinsic properties also affect interactions with immune cells. For example, disseminating small-cell lung cancer cells are more sensitive to NK cell–mediated elimination of cancer cells but less sensitive to elimination mediated by CD8^+^ or CD4^+^ T cells (82, 83). It remains unanswered whether other cells act in concert with NK cells to eliminate circulating cancer cells and whether the mechanisms of interactions between NK cells and disseminated cancer cells differ by tumor type.

## Dormant cancer cells resist NK cells to escape surveillance

The timing of metastatic development is also under intense study. Dormant cancer cells are strictly defined as cancer cells that are nonproliferating and have undergone G_0_ to G_1_ cell cycle arrest (84). Metastatic cancer cells can enter a state of dormancy to persist in distant organs and remain hidden from the immune system and clinically undetectable for multiple years before presenting as relapsed disease (85). Intrinsic features that allow cancer cells to enter and maintain a dormant state have now been defined (86). However, key questions remain, including how dormant metastatic cancer cells survive immune selection and evade NK cell cytotoxicity. Answering these questions could provide therapeutic targets for more effective adjuvant immunotherapies.

After isolating lung and breast metastatic dormant cells, Malladi et al. inoculated athymic mice and NOD/SCID gamma chain deleted (NSG) mice with latency competent cancer cells (LCCs) to assess the impact of immune selective pressure (87). Athymic mice lack T cells but have intact NK cells, while NSG mice lack both innate and adaptive immune compartments. While numbers of metastatic dormant cells were decreased in athymic mice, they observed that all NSG mice developed overt metastases. This result suggested that NK cells restricted the outgrowth of metastatic dormant cells as they exited quiescence, although it leaves unanswered whether other innate lymphoid cells could play a role. However, the study’s hypothesis was later confirmed with functional coculture experiments and transcriptomic analysis that revealed that quiescent dormant cells are resistant to NK cell cytotoxicity and downregulate receptors related to NK cell activation. However, the authors also reported downregulation of CD155, a ligand that binds to both DNAM-1, an NK cell activating receptor (88, 89), and the NK cell inhibitory receptor TIGIT (90). This observation highlights the complex interplay behind NK cell activating and inactivating signaling. How NK cells integrate the numerous and sometimes opposing signals to drive their dominant response is under active investigation. Metastatic dormant cells were observed to resist WNT activation through the autocrine expression of the Dickkopf Wnt signaling pathway inhibitor (DKK1) (87). They found that increased DKK1 expression correlates with increased NK cell activating receptor expression (87). Further, DKK1 knockdown increased the expression of NK cell activating ligands *Ulbp1*, *Ulbp2*, *Ulbp4*, and *Ulbp5* and death signaling receptors (87). In a colon cancer model, DKK2 inactivated NK cells through binding LRP5 and impeded the activity of STAT5 (91). Collectively, these studies provide a therapeutic opportunity to target DKKs in order to restore the antitumor function of NK cells.

**Figure 1:**
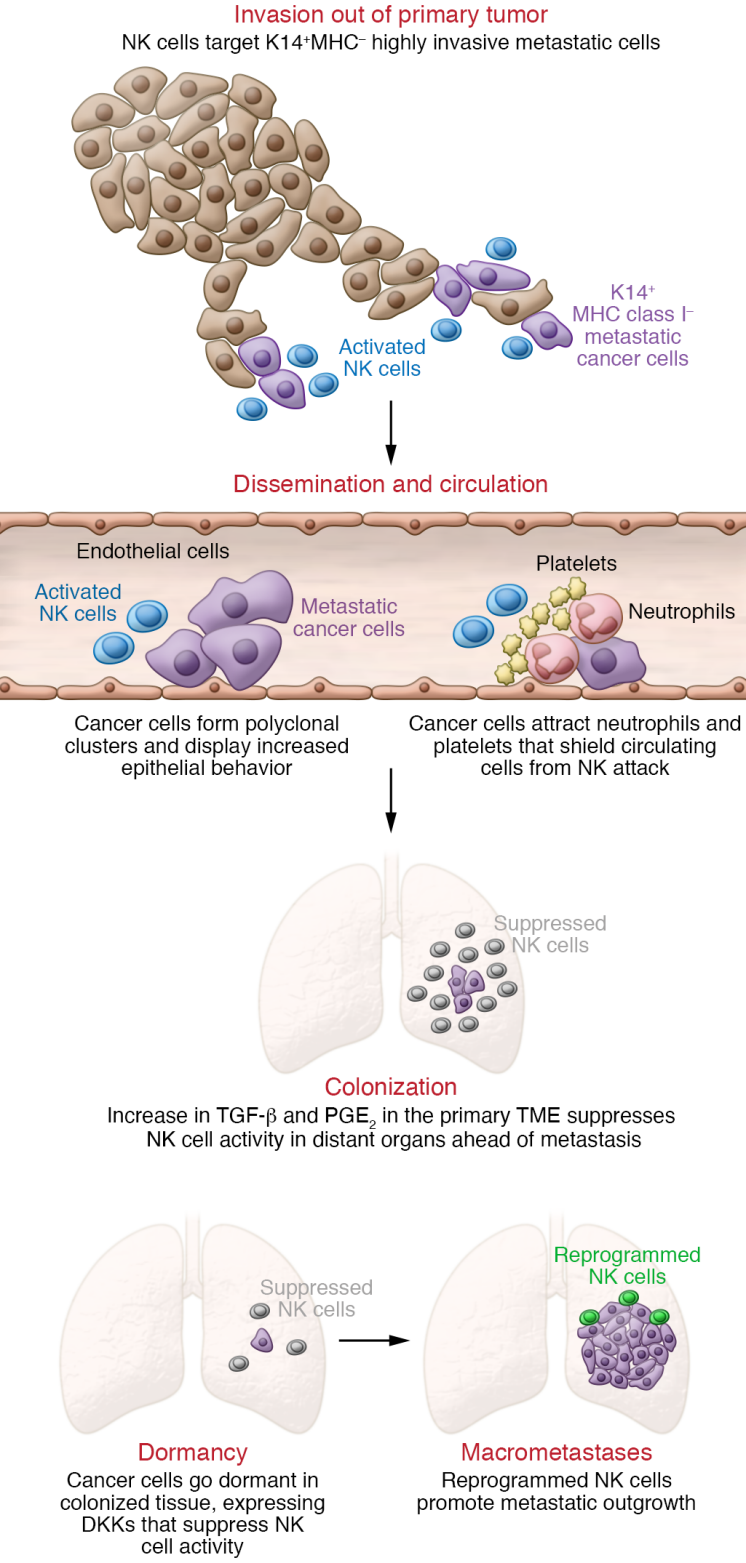
The varied roles of NK cells during metastasis. NK cell activity varies in different organs and at different stages of metastasis. Contributing to the different functions of NK cells are the changes within the cancer cell that occur at each stage. Initially NK cells control metastasis by targeting highly invasive metastatic cells that express K14 and lack MHC class I molecules (14). However, as cancer cells change their shape, features, and molecular composition during the process of metastasis, they can alter the function of NK cells as a mode of immune escape. As polyclonal clusters of metastatic cancer cells disseminate and circulate, they display increased epithelial behavior (55) and recruit neutrophils that shield them from NK cell attack (69). During colonization of a distal organ, increases in TGF-β (61) and PGE_2_ (65) in the tumor microenvironment (TME) inactivate NK cells. Dormant cancer cells in colonized tissue express DKKs to suppress NK cell activity (37, 40). Finally, NK cells undergo reprogramming to express inhibitory rather than activating receptors as metastatic cancer cells proliferate into macrometastases, and the reprogrammed NK cells can promote tumor growth (14).

Laughney et al. provided further evidence of how transitions out of metastatic dormancy increase sensitivity to NK cell cytotoxicity (92). Using single-cell transcriptional analysis, the authors discovered that, in lung cancer, metastatic lesions contain cancer cells that mirror the developmental continuum of stem to progenitor-like states of adult lung epithelial lineages. These genomic definitions mirrored functional transitions as dormant cells progressed to form macrometastatic lesions. Notably, the authors found that as cancer cells began to exit dormancy and enter a regenerating state, these cells had lower expression of genes related to MHC class I and higher expression of genes related to NK cell activating ligands, such as *ULBP*s and *RAET1* (92). However, as cancer cells began to proliferate into macrometastases, these NK cell activating signals were reversed. These findings suggest that the kinetics and specific functional properties of metastatic cancer cells dictate their sensitivity to NK cell–mediated immunoediting (Figure 1).

These studies help define an emerging field of metastatic dormancy. The characterization of this cell state continues to improve, across the stages of metastasis and under different therapeutic selection pressures (e.g., endocrine therapy in breast cancer). It will be interesting as new data emerge regarding whether metastatic dormant cells elicit a similar NK cell response regardless of tumor type or whether there is a specific NK cell response unique to each cancer. How these metastatic dormant cells emerge from dormancy, escape NK cell surveillance, and proliferate into clinically detectable disease will need further study.

## Cancer cells can reprogram NK cells to support metastases

Multiple immune and stromal cells have been shown to increase the metastatic potential of cancer cells and aid early dissemination of cancer cells, including macrophages (93), neutrophils (94–96), fibroblasts (97), platelets (98), and regulatory T cells (99). Less is known about whether cancer cells can reprogram NK cells to a pro-metastatic state. Our group recently showed that NK cells are the most abundant innate immune cell responding to K14-positive cancer cell clusters arriving in the lung (51). K14 is a basal epithelial marker that marks highly migratory cell populations in development and cancer (100). As discussed above, we previously defined the expression and requirement of K14 in these highly metastatic breast cancer cells, which lead collective invasion, systemic dissemination, and colonization of distant organs (63, 101). In studying how this subset of invasive cells evades immunosurveillance, we found that K14-positive cells did not express MHC class I molecules. MHC class I molecules are a major class of NK cell inhibitory signals (102), suggesting that these invasive cells respond to NK cell targeting. Next, to test how NK cells interact with metastatic cancer cells, we developed a novel NK cell–organoid ex vivo 3D coculture platform. These assays recapitulate NK cell–cancer cell interactions and allow us to observe in real time as NK cells induce apoptosis in cancer cells during invasion and colony formation. We found that NK cells specifically targeted K14-positive cells for cytotoxic activity, resulting in reduced collective invasion and metastatic colony formation (Figure 1).

Yet, despite the potent anti-metastatic effects of surveilling NK cells, metastases emerge in breast cancer patients. To address how NK cells are altered by cancer cells, we isolated NK cells that had been exposed to the tumor (tumor-exposed, or teNK, cells). We tested their function in our varied 3D coculture assays. To our surprise, teNK cells promoted colony formation over monoculture controls (51). These ex vivo findings were confirmed with in vivo adoptive transfer experiments with teNK cells. Our findings may help to explain clinical observations in melanoma and breast cancer that increased NK cell numbers do not always correlate with increased survival (103, 104).

To identify molecular strategies to reverse the metastasis-promoting effects of teNK cells, we performed transcriptomic analysis comparing teNK cells to healthy NK cells. Using live imaging, we observed that NK cells and metastatic cancer cells interact repeatedly. This led us to perform additional bioinformatics analyses, which revealed receptor-ligand pairs between K14-positive cells and teNK cells. To validate potential candidates, we tested blocking antibodies that target two identified inhibitory receptors highly expressed by teNK cells: TIGIT and KLRG1. Treatment with either anti-TIGIT or anti-KLRG1 neutralized the effect of teNK cells and reduced colony formation. In contrast, treatment with antibodies against programmed cell death protein 1 (PD-1) did not limit the colony-promoting effect of teNK cells. We found that DNA methyltransferases (*Dnmt1*, *Dnmt3a*, *Dnmt3b*) were highly expressed by teNK cells relative to healthy NK cells, suggesting that the reprogramming of NK cells by cancer cells is epigenetically controlled. Treatment with FDA-approved DNMT inhibitors also neutralized the teNK cell effect on colony formation. Combination therapy with both DNMT inhibitors and anti-TIGIT or anti-KLRG1 antibodies significantly reduced the number of colonies formed (51). An exciting extension of this work will be to determine which combination of epigenetic therapies and inhibitory receptor blockade can significantly restore and sustain NK cell cytotoxicity in metastatic models.

Our results show that NK cells are capable of considerable functional plasticity in response to cues from the cancer cell. Several other groups have shown that NK cells can be induced to secrete various factors to promote the metastatic niche. For example, TGF-β is an important immunomodulator of the immune microenvironment (105) that can reduce the activation, proliferation, and cytolytic activity of NK cells through the mTOR pathway (106). Using mouse models of impaired or constitutively active TGF-β signaling, Gao et al. found that TGF-β is sufficient to convert classical NK cells into populations of type 1 innate lymphoid cells (ILC1s) (107). The authors found that NK cells that were CD49a^–^CD49b^+^ could be converted into an intermediate form of ILC1 (defined by CD49a^+^CD49b^+^) or classical ILC1s (defined as CD49a^+^CD49b^–^). These NK cell–derived ILC1s had higher gene expression of *Ctla4* and *Lag3*, markers of immune exhaustion. The authors observed in functional metastasis models that intermediate ILC1s and ILC1s do not impair metastatic development. Instead, these cells express high levels of TNF, which the authors postulate is one of several factors that allow ILC1s to contribute toward a pro-tumorigenic microenvironment (107). These findings are supported by other studies showing that STAT5-deficient NK cells secrete VEGFA, which in turn stimulates endothelial cell growth and tumor angiogenesis (108). So just as cancer cells are able to co-opt macrophages and neutrophils, they can also shift NK cells in favor of tumor promotion.

Our findings also support a role for inflammatory signaling in cancer progression (109). It will therefore be important to understand the contributions of chronically activated or inactivated NK cells. For example, NKG2D is a known activating immunoreceptor in T cells and NK cells (110). However, using a model of a chemically induced liver cancer in NKG2D wild-type or knockout mice, Sheppard et al. showed how chronically activated CD8^+^ T cells expressing NKG2D contribute to liver cancer development and a proinflammatory state within the tumor and surrounding tissues (111). The authors propose a model that NKG2D can act early in precancerous lesions to activate immune effector responses like NK cells to eliminate the tumor (111). However, in doing so, this process allows for the development of a proinflammatory environment that becomes tumor promoting (112). While these observations were observed in primary tumor development, distant-site metastases often develop under proinflammatory conditions (113), and inactivated NK cells or chronically stimulated NK cells could play a role in supporting metastatic outgrowth.

## The metastatic niche suppresses NK cell cytotoxicity

Beyond inactivating NK cytotoxic function, we are beginning to understand that cancer cells can alter the tumor microenvironment (TME) to support metastatic outgrowth. A growing body of literature has shown that cancer cells can secrete factors like VEGFA, angiopoietin-like ligands, chemokine C-C motif ligands, matrix metalloproteinases, IL-6, prostaglandin E_2_ (PGE_2_), and TGF-β that prepare distant microenvironments for colonization (refs. 114, 115, and Figure 1). These factors in the metastatic niche are also known to impair the function of NK cells. For example, cancer cells can secrete PGE_2_ to disrupt the NK cell–dendritic cell axis (116). Not only can secreted PGE_2_ directly reduce NK cell production of IFN-γ (117), it also impairs NK cell–mediated recruitment of dendritic cells and the responsiveness of dendritic cells to these chemokines.

**Figure 2:**
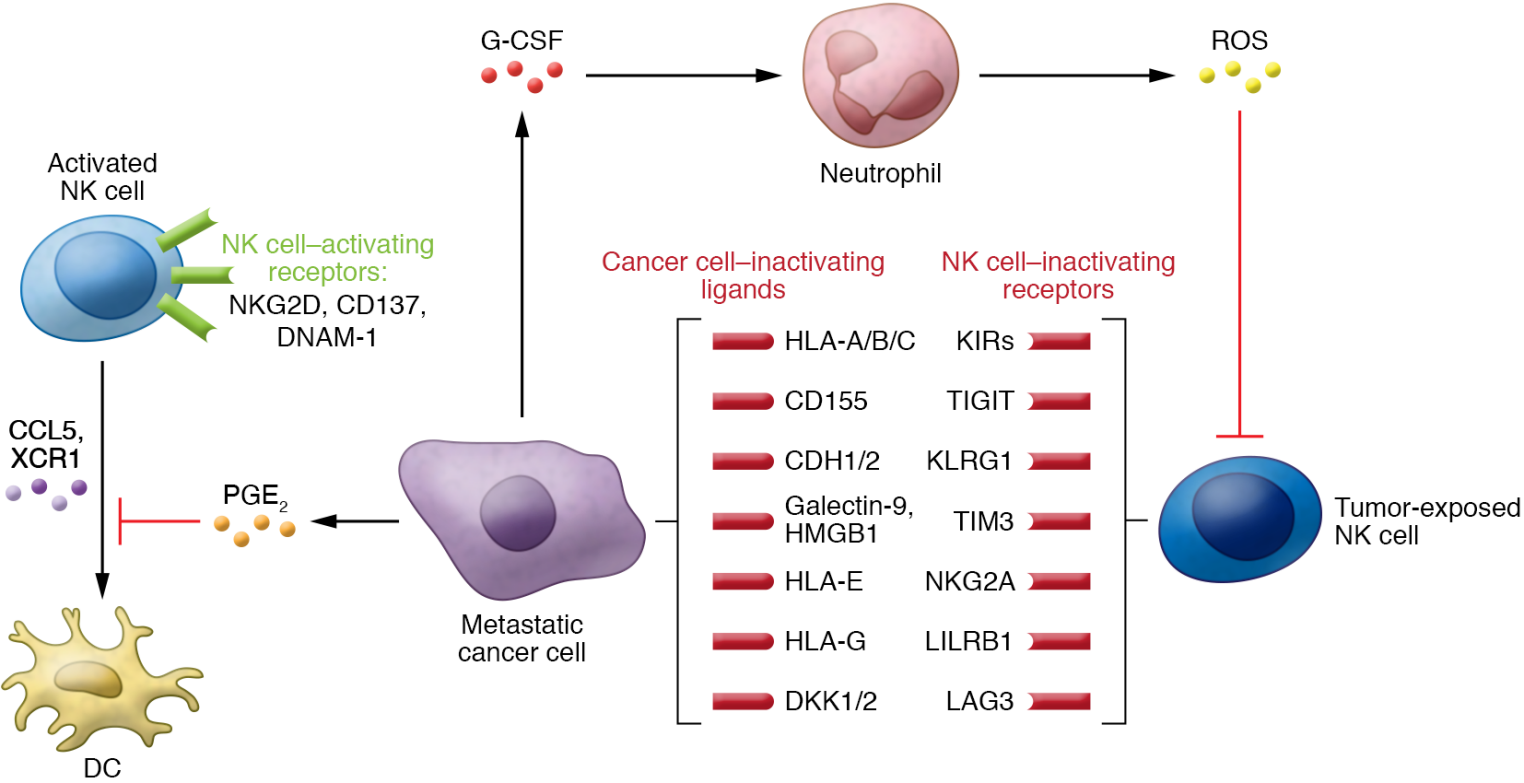
Signaling interactions between NK cells and cancer cells in the tumor microenvironment. Natural killer cells within the tumor microenvironment are governed by a series of signals from cancer cells and from other immune cells that are present. During metastasis, exposure to cancer cells can alter these signals to activate or inactivate the NK cells or alter them toward a tumor-promoting phenotype.

Aside from containing NK cell–suppressing signals, the metastatic niche can support disease progression in a variety of ways (Figure 2). These range from providing structural attachment for anchorage to stimulating regulatory immune cells to inhibiting antitumor effector cells (118). For example, using in vivo and in vitro tri-culture models, Li et al. recently found that metastatic cancer cells secrete G-CSF to attract neutrophils that are anti-metastatic in NK cell–deficient mice (119). However, in the presence of NK cells, G-CSF attracts neutrophils that suppress NK cell cytotoxic activity through ROS signaling, and ultimately enhance metastatic outgrowth. These data are consistent with other observations of neutrophils suppressing NK cell activity (120). In this study, Spiegel et al. used a syngeneic mouse model of breast cancer to show that metastatic cells can co-opt neutrophils to increase both the dissemination of cancer cells out of the primary tumor and their subsequent intravasation into lung vasculature (120). Using in vivo NK cell–depleting antibodies and NK cell–responsive cell lines, they showed that neutrophils were able to shield intraluminal metastatic cancer cells from NK cell clearance (ref. 120 and Figure 1). Neutrophils also prevented NK cells from achieving functional activation. Interestingly, these results also suggest that NK cells respond rapidly to clear intraluminal cancer cells within 24 hours. Beyond this time point, NK cells had minimal impact on metastatic lesion development. Other cells, such as platelets, have been shown to shield cancer cells from NK cell cytotoxicity, and platelet depletion led to decreased tumor seeding of distant organs (121). Follow-up studies reported that Gαq, a protein critical to platelet activation, was necessary for the establishment of lung metastases (122). When NK cells were depleted in *Gαq^–/–^* mice, there was no change in the number of lung metastases versus control mice depleted of NK cells (122). These results suggest that platelet function itself is required for their tumor-protective ability and not merely provision of a physical barrier.

Immunosuppressive cells, such as regulatory T cells (Tregs), have been observed at the metastatic site and can inhibit NK cell activity (123). In a model of melanoma metastasis, Wang et al. showed that classical Tregs can suppress NK cell activity through direct cell-cell contact mediated by Qa-1/NKG2A engagement (124). Tregs in general have been shown to suppress NK cells through direct physical interactions via β-galactoside–binding protein (125) or through secreted factors like IL-37 (126). Interestingly, IL-37 has been shown to decrease metastasis in several models of cancer (127, 128), and requires further scrutiny on its immunomodulatory impact in the metastatic microenvironment.

Other immunoregulatory cells that suppress NK cell cytotoxicity through physical interactions are tumor-associated macrophages (TAMs). While patrolling monocytes can contribute to the activation of NK cells to target metastatic cancer cells in the lung (129), TAMs have also been shown to attenuate NK cell function through CD48 expression in hepatocellular carcinomas (130). In gastric cancer, TAMs can also inhibit the antitumor effects of NK cells through the secretion of TGF-β1 (131). Myeloid-derived suppressor cells (MDSCs) can also act to inhibit NK cells’ suppression of metastasis (132). For example, one study documented correlations between increased numbers of MDSCs at the metastatic site, decreased NK cell activity, and increased lung metastases during pregnancy (133). At the liver, Li et al. used metastatic mouse models to show that MDSCs directly suppress hepatic NK cell production of IFN-γ through membrane-bound TGF-β. Knockout of Smad3 in hepatic NK cells eliminated the ability of MDSCs to impair NK cell cytotoxicity (134). Understanding how other immune cells in the metastatic cascade regulate NK cell function will be especially important in applying NK cell–directed therapies to treat metastatic disease.

## Translating NK cell biology during metastasis into therapeutics

Metastatic disease has also historically been difficult to treat because the biology of the metastatic cancer cell is plastic and context dependent and TME composition is stage specific. Also contributing to the difficulty of developing new immunotherapies are the unique off-target effects that can occur. For example, attempts to modulate IL-2 in the TME can improve cytotoxic function of specific effector immune cells but can also increase the immunosuppressive function of other immune cells (135). Here we review NK cell–directed therapies that target metastasis or are administered in the metastatic setting, while we redirect readers to other recent reviews that have extensively covered specific classes of NK cell–directed therapies (3, 22, 136).

A consistent theme across preclinical studies is that an optimal therapeutic window exists to achieve maximal NK cell anti-metastatic activity. NK cell abundance and activity appear to be highest before the development of macrometastases, potentially avoiding the inhibitory signals expressed by larger lesions and co-option. These observations suggest that NK cell–directed therapies would be most potent in the adjuvant setting; specifically, NK cells may be most effective when they are being used to target disseminated cancer cells in circulation or those that have been deposited in distant organs. An analogous example is the use of immune checkpoint blockade in the neoadjuvant and adjuvant setting in breast cancer, which resulted in improved event-free survival rates (137, 138).

Yet in order for NK cell–directed therapies to be most effective in the treatment of patients with metastatic disease, it will be important to understand the specific signaling between distant-site metastatic cancer cells and NK cells. As “omics” approaches become more refined at the single-cell level, we can leverage network-level analyses to provide some early clues (139). Identification of the main communicating signals between metastatic cancer cells and NK cells at the distant site among the varied modes of communication will be critical. Therapies inhibiting checkpoint receptors that diminish T cell activity from engaging ligands expressed by cancer cells have proven to be very effective clinically. Multiple monoclonal antibodies directed at blocking inhibitory signaling on NK cells are being tested in early-phase clinical trials, often in the metastatic setting. These agents include monalizumab (blocking NKG2A activity; ref. 140), lirilumab (blocking the family of KIR2D; ref. 141), tiragolumab (anti-TIGIT blocking antibody; ref. 142), and anti-LAG3 agents like IMP321 and relatlimab (143). Monalizumab has entered phase III clinical trials (ClinicalTrials.gov NCT04590963) after a successful phase Ib/II trial (144). As T cells and NK cells can both express many of these receptors, further investigation is needed to determine the contribution of NK cells to the observed effects at metastatic sites.

Therapies directed at harnessing NK cells to control metastatic cancer cells at distant sites will also need to contend with an immunosuppressive TME. There has been a resurgence of interest in blocking or removing TGF-β signaling from the TME (145), which has immunosuppressive effects on multiple cytotoxic immune cells, including NK cells. An interesting approach to remove TGF-β is to use a bifunctional fusion protein that contains the extracellular domain of TGF-βRII receptor fused to a human IgG1 monoclonal antibody blocking PD-L1. Bintrafusp alfa uses this technology and simultaneously eliminates TGF-β from the TME while blocking the PD-(L)1 pathway (146, 147). Interim analysis of a phase I clinical trial (NCT02517398) investigating its use in non–small cell lung cancer demonstrated durable responses (148). A follow-up phase III study will compare bintrafusp alfa directly with the anti–PD-1 agent pembrolizumab in patients with PD-L1–expressing advanced non–small cell lung cancer (NCT03631706). While these therapies are not specific to NK cells, one could envision a path forward using NK cell–specific targets while simultaneously removing immunosuppressive signaling at the metastatic site.

## Future directions

Cancer immunotherapies are rapidly evolving. NK cells provide another population of immune cells that can be used to treat metastatic disease. Advancing our understanding of how NK cells interact with metastatic cancer cells is critical to developing personalized NK cell–directed therapies. There is a pressing need to develop improved preclinical models that capture how cancer cells physically interact with NK cells throughout metastasis. Models focused on late-stage metastatic outgrowth as a single endpoint do not account for the numbers of NK cells that react to early metastases, the mechanisms NK cells use to eliminate metastatic tumor cells throughout the metastatic cascade, or which cancer cells NK cells target within the metastatic lesion. These modes of communication can include interactions through specific receptor-ligand binding or paracrine communication, such as through exosomes or secreted ligands, chemokines, and cytokines.

As we improve preclinical modeling, we can further define the kinetics and characteristics of NK cell plasticity. We can use these models to expand our understanding of which NK cell phenotype along a functional spectrum is most active against metastatic cancer cells and when their antitumor activity is maximized. The functional and molecular plasticity of NK cells has been observed in multiple settings, including normal physiology (149, 150), infection (151), and cancer (152). How these phenotypes exist within the metastatic cascade remains to be uncovered. New insights could help guide future NK cell–directed therapies. Despite the unanswered questions that remain for NK cell–based approaches, tremendous progress has been made in our fundamental understanding of NK cell biology during metastasis. We are poised to use this knowledge to deliver a next generation of immunotherapies for patients with metastatic disease.

## Version 1 03/15/2022

Electronic publication
